# Supplementary material for: The rational use of thromboprophylaxis therapy in hospitalized patients and the perspectives of health care providers in Northern Cyprus
Source: PLoS One. 2020 Jul 15;15(7):e0235495. doi: 10.1371/journal.pone.0235495 (PMC7363080; doi:10.1371/journal.pone.0235495)
Supplement: S1 Appendix — (DOCX) [file pone.0235495.s001.docx]

**Annexure-A**

**Patient Case Documentation Form**

Patient Name: _______________________ Room #:_______ Admission Date: / / Discharge date: __________

Age: ______ Gender: _____ Ht: _______ Wt: ______ Case: Cardio Pulmonary GI DM Other: _________

IBW:______ kg: Crt. Cl ____ ml/min.

CC: ______________________________________________________________________________________________

HPI:______________________________________________________________________________________________________________________________________________________________________________________________________________________________________________________________________________________________________________________________________________________________________________________________________________________________________________________________________________________________________

PMH:_________________________________________________________________________________________________________________________________________________________________________________________________________________________________________________________________________________________________

FH:_________________________________________________________________________________________________________________________________________________________________________________________________

SH: Smoking _________________________________ EtOH:____________ Other: ____________________________

Allergies: _________________________________________________________________________________________

Home Rx: _________________________________________________________________________________________ ______________________________________________________________________________________________________________________________________________________________________________________________________________________________________________________________________________________________________

Hospital Course (include any pertinent labs): _____________________________________________________________

__________________________________________________________________________________________________________________________________________________________________________________________________________________________________________________________________________________________________________________________________________________________________________________________________________________________________________________________________________________________________________________________________________________________________________________________________________________________________________________________________________________________________________________________________________________________________________________________________________________________________________________________________________________________________________________________________________________________________________________________________________________________________________________________________________________________________________________________________________________________________________________________________________________________________________________________________________________________________________________________________________________________________________________________________________________________________________________________________________________________________________________________________________________________________________________________________________________________________________________________________________________________________________________________________


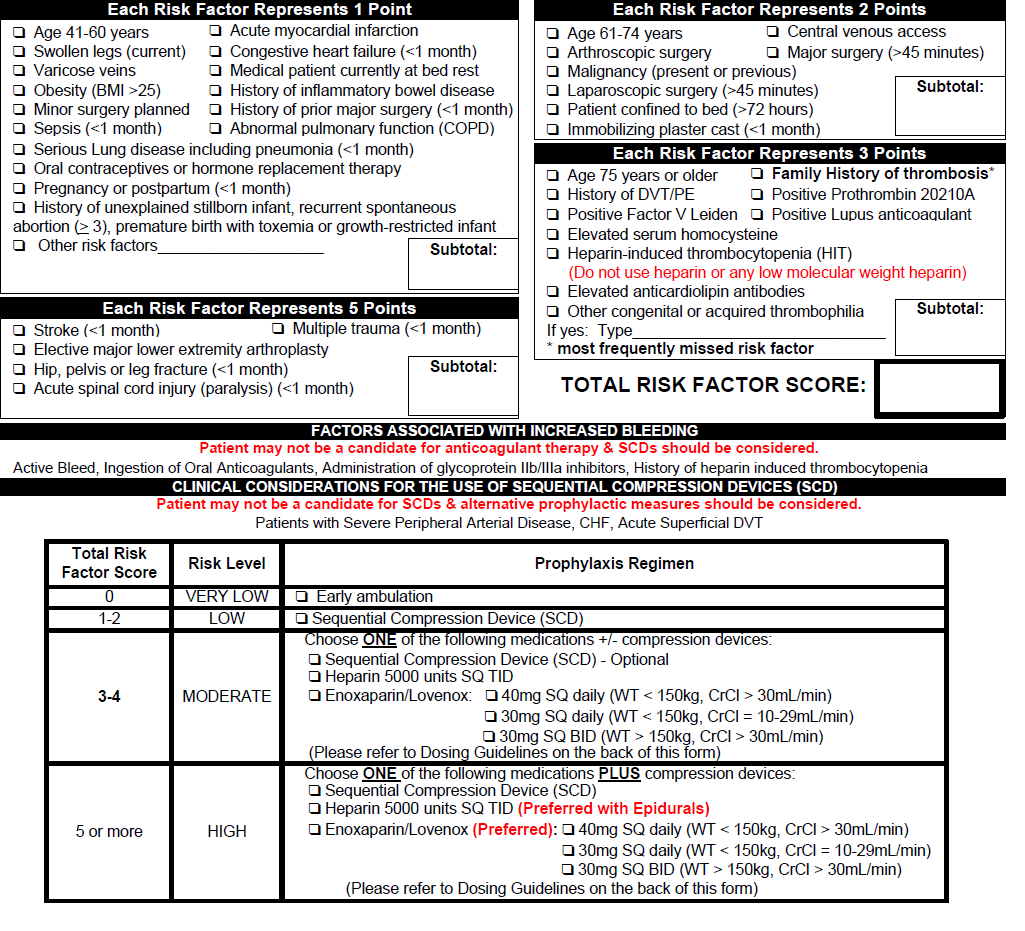


Current meds: _____________________________________ other medications during hospitalization: ____________

1. ______________________________________________ 1. _______________________________________________

2. ______________________________________________ 2. _______________________________________________

3. ______________________________________________ 3. _______________________________________________

4. ______________________________________________ 4. _______________________________________________

5. ______________________________________________ 5. _______________________________________________

6. ______________________________________________ 6. _______________________________________________

7. ______________________________________________ 7. _______________________________________________

8. ______________________________________________ 8. _______________________________________________

9. ______________________________________________ 9. _______________________________________________

10. _____________________________________________ 10. ______________________________________________

11. _____________________________________________11. _______________________________________________

12. _____________________________________________ 12. ______________________________________________

Overall assessment and Summary: __________________________________________________________________________________________________________________________________________________________________________________________________________________________________________________________________________________________________________________________________________________________________________________________________________________________________________________________________________________________________________________________________________________________________________________________________________________________________________________________________________________________________________________________________________________________________________________________________________________________________________________________________________________________________________________________________________________________________________________________________________________________________________________________________________________________________________________________________________________________________________________________________________________________________________________________________________________________________________________________
