# Supplementary material for: The rational use of thromboprophylaxis therapy in hospitalized patients and the perspectives of health care providers in Northern Cyprus
Source: PLoS One. 2020 Jul 15;15(7):e0235495. doi: 10.1371/journal.pone.0235495 (PMC7363080; doi:10.1371/journal.pone.0235495)
Supplement: S2 Appendix — (DOCX) [file pone.0235495.s002.docx]

**The rational use of thromboprophylaxis therapy in hospitalized patients and the perspectives of health care providers in Dr. Suat Gunsel Kyrenia University Hospital and Near East University Hospital in Northern Cyprus**

**Annexure B**

**1. Age**

a. ≤ 25

b. 26–30

c.  ≥ 31

**2. Educational degree**

a. Diploma

b. bachelor’s degree

c. master’s degree

d. PhD

**3. Gender**

a. Male

b. Female

**4. Years of nursing experience**

a. <5

b. 6–10

c. > 11

**5. Currently working unit**

a. Emergency care

b. Intensive care unit (ICU)

c. Internal medicine unit

d. Obstetrics/gynecology unit

e. Oncology unit

f. Surgical unit

g. Rehabilitation unit

h. Others

**6. Previous DVT education**

a. Yes

b. No

**7. Educational resource**

a. School

b. Courses

c. Web resources

d. Congress/conferences

e. In-service education

**8. Opinions on the quality of the DVT education**

a. Excellent

b. Very good

c. Good

d. Fair/poor

**9. Need for education on DVT**

a. Yes

b. No

**Nurses’ General Knowledge on DVT**

| **Statements on DVT** | **True/** | **Correct** | | | **Wrong answer / I don’t** | |
| --- | --- | --- | --- | --- | --- | --- |
|  | **False** | **answer** | |  | **know** |  |
|  |  |  |  |  |  |  |
|  |  | **N** |  | **%** | **N** | **%** |
|  |  |  |  |  |  |  |
| DVT occur as a result of stasis of blood |  |  |  |  |  |  |
| (venous stasis), vessel wall injury, and |  |  |  |  |  |  |
| altered blood coagulation. |  |  |  |  |  |  |
|  |  |  |  |  |  |  |
| Venous thromboembolism (VTE) is a fatal |  |  |  |  |  |  |
| complication of DVT. |  |  |  |  |  |  |
|  |  |  |  |  |  |  |
| VTE is a major cause of sudden death in |  |  |  |  |  |  |
| hospitalized patients. |  |  |  |  |  |  |
|  |  |  |  |  |  |  |
| Surgical patients are more prone than |  |  |  |  |  |  |
| medical patients to DVT/VTE. |  |  |  |  |  |  |
|  |  |  |  |  |  |  |
| DVT occurs most frequently in the veins |  |  |  |  |  |  |
| of the lower extremities. |  |  |  |  |  |  |
|  |  |  |  |  |  |  |
| Deep vein thrombosis also occurs |  |  |  |  |  |  |
| frequently in the upper limbs. |  |  |  |  |  |  |
|  |  |  |  |  |  |  |
|  |  |  |  |  |  |  |
|  |  |  |  |  |  |  |

**Nurses’ Knowledge on Risk Factors of DVT**

| **Statements on DVT Risk Factors** | **True/** | **Correct answer** | | **Wrong answer / I don’t** | |
| --- | --- | --- | --- | --- | --- |
|  | **False** |  |  | **know** |  |
|  |  |  |  |  |  |
|  |  | **N** | **%** | **N** | **%** |
|  |  |  |  |  |  |
| Prolonged immobilization predisposes to |  |  |  |  |  |
| DVT in hospitalized patients. |  |  |  |  |  |
| Indwelling intravenous devices such as |  |  |  |  |  |
| central venous catheters may predisposes to |  |  |  |  |  |
| DVT. |  |  |  |  |  |
|  |  |  |  |  |  |
| Paralysis, paresis, or recent plaster cast on |  |  |  |  |  |
| lower extremities may predispose to DVT . |  |  |  |  |  |
|  |  |  |  |  |  |
| Obesity may predisposes to DVT. |  |  |  |  |  |
|  |  |  |  |  |  |
| Low body mass index may predisposes to |  |  |  |  |  |
| DVT. |  |  |  |  |  |
|  |  |  |  |  |  |
| Advancing age may predisposes to DVT. |  |  |  |  |  |
|  |  |  |  |  |  |
| Previous DVT/VTE history may predisposes |  |  |  |  |  |
| to DVT. |  |  |  |  |  |
|  |  |  |  |  |  |
| There is no relationship between cancer or |  |  |  |  |  |
| cancer treatment and DVT/VTE. |  |  |  |  |  |
|  |  |  |  |  |  |
| Major surgery may predisposes to DVT. |  |  |  |  |  |
|  |  |  |  |  |  |
| Varicose veins may predispose to DVT. |  |  |  |  |  |
|  |  |  |  |  |  |
| Exercises may predisposes to DVT. |  |  |  |  |  |
|  |  |  |  |  |  |
| Trauma may predisposes to DVT. |  |  |  |  |  |
|  |  |  |  |  |  |
| Smoking may predisposes to DVT. |  |  |  |  |  |
|  |  |  |  |  |  |
| Alcohol may predisposes to DVT. |  |  |  |  |  |
|  |  |  |  |  |  |
| Cardiac diseases may predispose to DVT. |  |  |  |  |  |
|  |  |  |  |  |  |
| There is no relationship between respiratory |  |  |  |  |  |
| diseases and DVT. |  |  |  |  |  |
|  |  |  |  |  |  |
| Infections or inflammations may predispose |  |  |  |  |  |
| to DVT. |  |  |  |  |  |
|  |  |  |  |  |  |
| Pregnancy or post-partum may predispose to |  |  |  |  |  |
| DVT. |  |  |  |  |  |
|  |  |  |  |  |  |
| Oral contraceptives or hormone replacement |  |  |  |  |  |
| therapy may predispose to DVT. |  |  |  |  |  |
|  |  |  |  |  |  |
| There is no relationship between family |  |  |  |  |  |
| history of DVT/VTE and DVT. |  |  |  |  |  |
|  |  |  |  |  |  |
|  |  |  |  |  |  |
|  |  |  |  |  |  |

**Nurses’ Knowledge on Prevention of DVT**

| **Statements on DVT Prevention** | | **True/** | **Correct answer** | | **Wrong answer / I don’t** | |
| --- | --- | --- | --- | --- | --- | --- |
|  |  | **False** |  |  | **know** |  |
|  |  |  | **N** | **%** | **N** | **%** |
|  | |  |  |  |  |  |
| Foot and leg exercises may prevent | |  |  |  |  |  |
| DVT. |  |  |  |  |  |  |
|  | |  |  |  |  |  |
| Elevating legs is necessary to prevent | |  |  |  |  |  |
| DVT/ VTE. |  |  |  |  |  |  |
|  | |  |  |  |  |  |
| Early ambulation after surgery may | |  |  |  |  |  |
| prevent DVT development. | |  |  |  |  |  |
|  | |  |  |  |  |  |
| Bed rest is necessary after major | |  |  |  |  |  |
| surgery to prevent DVT. | |  |  |  |  |  |
|  | |  |  |  |  |  |
| Heparin or low molecular weight | |  |  |  |  |  |
| heparin (LMWH) may prevent DVT | |  |  |  |  |  |
| development. |  |  |  |  |  |  |
|  | |  |  |  |  |  |
| Fluid restriction is necessary to prevent | |  |  |  |  |  |
| DVT. |  |  |  |  |  |  |
|  |  |  |  |  |  |  |
| Elastic compression | stockings may |  |  |  |  |  |
| prevent DVT development. | |  |  |  |  |  |
|  | |  |  |  |  |  |
| The use of intermittent pneumatic | |  |  |  |  |  |
| compression devices | may prevent |  |  |  |  |  |
| DVT development. |  |  |  |  |  |  |
|  |  |  |  |  |  |  |
|  |  |  |  |  |  |  |
|  |  |  |  |  |  |  |

**Practices of Nurses on DVT Prevention**

|  |  |  |  | **Always** | |  | **Sometimes** | |  | **Never** | | |  |
| --- | --- | --- | --- | --- | --- | --- | --- | --- | --- | --- | --- | --- | --- |
| **DVT PreventionPractices** | |  |  |  |  |  |  |  |  |  |  |  |  |
|  |  |  |  | **N** | **%** |  | **N** | **%** |  | **N** |  | **%** | |
|  | | | |  |  |  |  |  |  |  |  |  | |
| Providing information to patients and/or relatives | | | |  |  |  |  |  |  |  |  |  | |
| about risks and prevention of DVT. | | |  |  |  |  |  |  |  |  |  |  |  |
|  | | | |  |  |  |  |  |  |  |  |  | |
| Encouraging patients to do foot and leg exercises by | | | |  |  |  |  |  |  |  |  |  | |
| themselves or relatives help if patients are unable to | | | |  |  |  |  |  |  |  |  |  |  |
| do so. |  |  |  |  |  |  |  |  |  |  |  |  |  |
|  | | | |  |  |  |  |  |  |  |  |  | |
| Encouraging early ambulation surgical of patients. | | | |  |  |  |  |  |  |  |  |  | |
|  | | | |  |  |  |  |  |  |  |  |  | |
| Assessing the DVT risks of patients the regularly. | | | |  |  |  |  |  |  |  |  |  | |
|  | | | |  |  |  |  |  |  |  |  |  | |
| Administering anticoagulants as preventive in clinic. | | | |  |  |  |  |  |  |  |  |  | |
|  | | | |  |  |  |  |  |  |  |  |  | |
| Monitoring the side effects of the anticoagulants. | | | |  |  |  |  |  |  |  |  |  | |
|  | | |  |  |  |  |  |  |  |  |  |  | |
| Educating the patients on anticoagulants. | | |  |  |  |  |  |  |  |  |  |  | |
|  | | |  |  |  |  |  |  |  |  |  |  | |
| Educating the patients to avoid injury. | | |  |  |  |  |  |  |  |  |  |  | |
|  | | |  |  |  |  |  |  |  |  |  |  | |
| Encouraging patients to do elevate legs. | | |  |  |  |  |  |  |  |  |  |  | |
|  | | |  |  |  |  |  |  |  |  |  |  | |
| Educating the patients on sufficient fluid | | | intake. |  |  |  |  |  |  |  |  |  | |
|  | | | |  |  |  |  |  |  |  |  |  | |
| Using of the graduated compression stockings. | | | |  |  |  |  |  |  |  |  |  | |
|  | | | |  |  |  |  |  |  |  |  |  | |
| Teaching the patients about proper use of graduated | | | |  |  |  |  |  |  |  |  |  | |
| compression stockings. | |  |  |  |  |  |  |  |  |  |  |  |  |
|  | | | |  |  |  |  |  |  |  |  |  | |
| Assessing the patients regularly for signs and | | | |  |  |  |  |  |  |  |  |  | |
| symptoms of DVT/VTE. | |  |  |  |  |  |  |  |  |  |  |  |  |
|  |  |  | |  |  |  |  |  |  |  |  |  |  |
