# Supplementary material for: The rational use of thromboprophylaxis therapy in hospitalized patients and the perspectives of health care providers in Northern Cyprus
Source: PLoS One. 2020 Jul 15;15(7):e0235495. doi: 10.1371/journal.pone.0235495 (PMC7363080; doi:10.1371/journal.pone.0235495)
Supplement: S3 Appendix — (DOCX) [file pone.0235495.s003.docx]

**The rational use of thromboprophylaxis therapy in hospitalized patients and the perspectives of health care providers in Dr. Suat Gunsel Kyrenia University Hospital and Near East University Hospital in Northern Cyprus**

**Annexure-C**

**Questionnaire for evaluation of surgeon’s and physician’s knowledge:**

**1. Which one of the following variable had more probability of risk for DVT?**

a. males

b. females

c. person less than 30 years old

d. person more than 40 years old

**2. Which one is correct?**

a. General anesthesia dose not increased DVT risk

b. Any duration for general anesthesia increased risk of DVT

c. General anesthesia only in abdominal or pelvic surgery raised risk of DVT

d. General anesthesia with more than 30 minute duration increased DVT risk

**3. Which one had more incidence of DVT during surgery?**

a. obese patient

b. surgery for malignancy

c. old age

d. pelvic surgery

**4. What is the most important mechanism or mechanisms of DVT risk during surgery?**

a. hypercoagulability

b. stasis

c. vascular injury

d. all of them.

**5. Which one is correct?**

a. DVT of thigh had 50% chance of pulmonary embolism.

b. Calf DVT had 40% probability of pulmonary embolism.

c. Proximal extenton of DVT decrease risk of pulmonary embolism.

d. DVT is a most common source of pulmonary embolism.

**6. Which one is not risk factor of DVT?**

a. Cardiac failure

b. peripartom state

c. OCP consumption

d. Surgery duration with less than 30 minute

**7. Which one of the following statements reflects the outcome of DVT without treatment?**

a. proximal extension

b. limitation by fibrinolysis or organization in calf DVT

c. embolisation risk increased

d. all of above

**8. Which one has not applicable for DVT prophylaxies during surgery?**

a. Intermittent pneumatic compression

b. low dose heparin

c. warfarin with INR of 2.5-3

d. elastic stocking

**9. Selection of DVT preventive measures determined by**

a. number of risk factors

b. type of surgery

c. kind of anesthetic drug

d. a and b

**10. In pulmonary thromboembolism which one is not correct?**

a. most common cause of preventable mortality in hospital

b. DVT is the most common source

c. most common cause of cyanosis in surgery

d. most of them have normal CXR

**11. Diagnosis and beginning of therapy for pulmonary thromboembolism is based on**

a. clinical criteria

b. simple hematologic tests

c. sophisticated imaging

d. clinical suspicious is enough

**12. DVT ocur as a result of stasis of blood (venous stasis), vessel Wall injury, and altered blood coagulation.**

a. True

b. False

**13. Venous thromboembolism (VTE) is a fatal combination of DVT.**

a. True

b. False

**14. VTE is a major cause of sudden death in hospitalized patients.**

a. True

b. False

**15. Surgical patients are more prone than medical patients to DVT/VTE.**

a. True

b. False
