# Supplementary material for: The rational use of thromboprophylaxis therapy in hospitalized patients and the perspectives of health care providers in Northern Cyprus
Source: PLoS One. 2020 Jul 15;15(7):e0235495. doi: 10.1371/journal.pone.0235495 (PMC7363080; doi:10.1371/journal.pone.0235495)
Supplement: S4 Appendix — (DOCX) [file pone.0235495.s004.docx]

**The rational use of thromboprophylaxis therapy in hospitalized patients and the perspectives of health care providers in Dr. Suat Gunsel Kyrenia University Hospital and Near East University Hospital in Northern Cyprus**

**Annexure-D**

**Attitude questions for physicians/surgeons**

| **Attitude statements Strongly Agree Strongly Disagree Disagree Neutral Agree** | | | | | |
| --- | --- | --- | --- | --- | --- |
| 1. I believe that Doppler  sonography (sensitive and  objective tests) is necessary to  screen for post-surgical DVT  in patients. |  |  |  |  |  |
| 2. I believe that an assessment  of DVT risk factors is  necessary prior to surgery. |  |  |  |  |  |
| 3. I believe that the  prevention/prophylaxis of  DVT is necessary prior to  surgery. |  |  |  |  |  |
| 4. I believe that educating  patients regarding preventive  measures of DVT is  necessary. |  |  |  |  |  |
| 5. I believe that nurses require  training to in methods to  prevent DVT. |  |  |  |  |  |
| 6. I believe that the prevention  of DVT with low-dose  heparin is irrational before  surgery. |  |  |  |  |  |
