# Supplementary material for: The rational use of thromboprophylaxis therapy in hospitalized patients and the perspectives of health care providers in Northern Cyprus
Source: PLoS One. 2020 Jul 15;15(7):e0235495. doi: 10.1371/journal.pone.0235495 (PMC7363080; doi:10.1371/journal.pone.0235495)
Supplement: Attachment — Submitted filename: Response to Reviewers.docx [file pone.0235495.s005.docx]

**Response to Reviewers Comments**

Dear reviewers and academic editor, thanks much for your time and fruitful comments on the article “The rational use of thromboprophylaxis therapy in hospitalized patients and the perspectives of health care providers in Northern Cyprus” Your review and comments would further help enhance the readability and the delivering of the message this article carries.

In the following few lines we would like to share our responses and describe the enhancement we made on the article following your comments and recommendations.

**Regarding Editor Comments:**

**Point 1:** Please ensure that your manuscript meets PLOS ONE's style requirements, including those for file naming.

**Response 1:** Thanks for the comment, we corrected page style, manuscript, references as well as file naming according to PLOS one instructions.

**Point 2:** Please provide additional details regarding participant consent. In the ethics statement in the Methods and online submission information, please ensure that you have specified (a) whether consent was informed and (b) what type you obtained (for instance, written or verbal, and if verbal, how it was documented and witnessed). If your study included minors, state whether you obtained consent from parents or guardians. If the need for consent was waived by the ethics committee, please include this information.”

**Response 2:** Thanks for the comments. The “Ethical Consideration” section was revised and corrected as follows:

“The study protocol was approved on 29th March 2018 by the Institutional Review Board (IRB) of Near East University (YDU/2018/56-530) and assigned as an observational study. A written consent form was signed by healthcare providers upon their participation in the study. A verbal consent was obtained from patients and recorded on data collection form upon their follow-up interviews”. Please see page 6 lines 137-141.

**Point 3:** Please include additional information regarding the survey or questionnaire used in the study and ensure that you have provided sufficient details that others could replicate the analyses. For instance, if you developed a questionnaire as part of this study and it is not under a copyright more restrictive than CC-BY, please include a copy, in both the original language and English, as Supporting Information. Please also include the exact number of individuals involved in the pilot testing of the questionnaire.

**Response 3:** Thanks for the comments. Copy of the questionnaire is attached in the supporting documents. The number of individuals involved in the pilot study was written as follows ( page 6 lines 132-133):

A pilot study was performed that targeted 10 to 15% of the study population, e.g. patients (n=35), nurses (n=40) and physicians (n= 15).

**Point 4:** Please refer to any sample size calculations performed prior to participant recruitment. If these were not performed, please justify the reasons. Please refer to our statistical reporting guidelines for assistance.

**Response 4:** Thanks for the comment**.** Statistical analysis section of the manuscript was reviewed and corrected as follows:

“Raosoft software version 2.3 (Raosoft. Inc., Seattle, USA) was used to calculate the minimum sample size required for the study. Assuming a 95% confidence level, a 5% margin of error and a 50% response distribution, at least 172 patients were needed to participate in the study out of 310 admitted to the hospital during the study duration. While 98 physicians and 169 nurses were required as minimum required sample out of 130 physicians and 300 nurses providing care at the two hospitals involved in the study”.

Please check Statistical section, page 7 lines 147-152.

Thanks much for your effort and contribution to our work

**Regarding first reviewer comments:**

**Point 1**: What is the aim of the study? There are more than one version.

Response 1: Thanks for the recommendation. The aim was revised and rewritten accordingly.

This study aims to evaluate the current thromboprophylaxis practice and management of hospitalized patients in different clinics along with the assessment of health care providers (HCPs) knowledge, and attitudes regarding DVT prophylaxis at two tertiary hospitals.

Please check aim section, page 1 lines 20-23.

**Point 2:** Where was the study done?

**Response 2:** Thanks for the comment, study setting was revised and rewritten as follows:

The study was conducted in the general wards of two tertiary university hospitals, NEU hospital in Nicosia and KUH in Kyrenia of Northern Cyprus.

Please check Study Setting and Subjects: page 4 lines 74-76.

**Point 3:** The inclusion criteria were acute and chronically ill medical patients…. But table 1 shows patients from diverse wards – including orthopaedic and surgery? This needs to be described.

**Response 3:** Thanks for your recommendation, the inclusion criteria is corrected and rewritten as follow:

The inclusion criteria were acute and chronically ill hospitalized patients for whom complete medical records were available. Please see page 4 line 78-79.

**Point 4:** Although the authors reported having followed up participants to asses for possible complications, the development of post-discharge complications (deep venous thrombosis signs and symptoms, pulmonary embolism or adverse effects of medications) was not obvious in the manuscript.

**Response 4:** Thanks. Paragraph 3 in results section, page 11, Line 188-191 states that no major complications were recorded as follows:

No major complications (e.g. gastrointestinal or retroperitoneal bleeding, thrombocytopenia or fatal pulmonary emboli) were recorded during hospitalization and post-hospitalization follow-up visits.

**Point 5:** The statement “The average length of hospitalization was 12.48 days (median of 15 days)” is unclear. Did the authors report the mean (average – 12.48) and median (15) at the same time? Why were the two measures reported together?

**Response 5:** Thanks much. The sentence was revised, corrected and rewritten as follows:

The median length of hospitalization stay was median (15) and IQR (29.75-7.00).

Please check page 7 lines 162-163.

**Point 6:** Table 2 describes "major" risk factors. Why are these risk factors called major? I am not sure if the term major is used in the Caprini score.

**Response 6:** Thanks much. Yes, that’s true, the sentence is corrected and rewritten as:

The most common risk factors identified in the sampled patients included an age of 41-60 years (26.1%), obesity (BMI>25) (21.1%), patients who were confined to bed for more than 3 days (100%), an age of 61-74 years (37.8%), and an age ≥75 years (28.3%). The distribution of risk assessment items and risk factors among sampled patients is shown in Table 2.

please check page 8 lines 170-173.

**Point 7**: In some areas, absolute numbers and percentages are given without reference to what is the denominator. A reader would struggle to identify the denominator. One of these sentences includes "Four of these patients (30.76%) developed 6 minor complications. Anticoagulation therapy was stopped in 2 patients (50%)”

Response 7: Thanks, it is reviewed and rewritten accordingly please check page 10 lines 184-185.

Only four of these 13 patients developed minor complications while anticoagulation therapy was stopped in 2 patients. .

**Point 8:** The importance of table 3 is unclear. What does table 3 answer? It looks redundant if there is nothing exclusive that it adds to the study.

Response 9: Very true. it is reviewed, table 3 is deleted and information is rewritten accordingly

Eight of these patients developed signs and symptoms of DVT (e.g. Warm feelings of legs in 4 patients, Leg swelling in 3 patients, etc.) please check page 11 lines 193-195.

**Point 9: There are several areas where authors used the word "female" to refer to "females."**

Response 9: Thanks for your attention. The word female is changed to females in the manuscript.

Thanks a lot for your effort and contribution to our work.

**Regarding Second reviewer comments:**

**Point 1:** Study Setting and Subjects: “All inpatients admitted between 01 April 2018 and 01 July 2018 who met the inclusion criteria were included in the analysis”. The authors need to clarify and edit the exclusion criteria stated. (lines 87 to 89) as the language is unclear

**Response 1:** Thanks. According to reviewer suggestions, the exclusion criteria was rewritten as follows:

Patients having age <18 years, superficial vein thrombosis, or any contraindications for DVT prophylaxis and patients who had deep venous thrombosis prophylaxis within the last month were excluded from the analysis. See page 4 line 80-82.

**Point 2:** Did the authors have a predetermined minimum required sample size for their analysis plan?

**Response 2:** Thanks for your comment. Yes, sample size evaluation was mentioned as follows:

**Statistical analysis section of the manuscript was reviewed and corrected as follows:**

“Raosoft software version 2.3 (Raosoft. Inc., Seattle, USA) was used to calculate the minimum sample size required for the study. Assuming a 95% confidence level, a 5% margin of error and a 50% response distribution, at least 172 patients were needed to participate in the study out of 310 admitted to the hospital during the study duration. While 98 physicians and 169 nurses were required as minimum required sample out of 130 physicians and 300 nurses providing care at the two hospitals involved in the study”.

Please check Statistical section, page 7 lines 147-152.

**Point 3:** The authors also need to clarify at what point data was initially collected-did this happen while patient was still on the ward or after they had been discharged. Also, it’s not clear what data was collected from the patient’s chart/record.

**Response 3:** Thanks for your comment. Data collected is mention in page 4 line 85-90 as follows:

“Demographic information, the presence of other DVT risk factors, any prophylaxis treatment administered for VTE, and a history of signs and symptoms of PE or DVT or anticoagulant complications documented in the patient files during hospitalization were collected. Patients were also assessed for possible complications by the research team during their follow-up visit two weeks after hospitalization to record any deep venous thrombosis signs and symptoms, pulmonary embolism or adverse effects of medications.”

**Point 4:** Ethical consideration: The authors clearly state that they received IRB ethics approval. However, it is also essential to state whether study participants provided written or verbal informed consent prior to participation and if not, state justification for waiver of this requirement.

**Response 4:** The “Ethical Consideration” section was revised and corrected as follows:

“The study protocol was approved on 29th March 2018 by the Institutional Review Board (IRB) of Near East University (YDU/2018/56-530) and assigned as an observational study. A written consent form was signed by healthcare providers upon their participation in the study. A verbal consent was obtained from patients and recorded on data collection form upon their follow-up interviews”. Please see page 6 lines 137-141.

**Point 5:** The authors need to mention the number of patients admitted during the 3-month study period from which they selected participants as stated in the methods section.

**Response 5:** Thanks for your comments. This was mention in page 4 line 76-78 as follows:

All patients admitted to the hospital and meet the inclusion criteria were invited to participate (n = 310).

Also, minimum Sample size required was mention in statistical analysis section as follows:

“Raosoft software version 2.3 (Raosoft. Inc., Seattle, USA) was used to calculate the minimum sample size required for the study. Assuming a 95% confidence level, a 5% margin of error and a 50% response distribution, at least 172 patients were needed to participate in the study out of 310 admitted to the hospital during the study duration. While 98 physicians and 169 nurses were required as minimum required sample out of 130 physicians and 300 nurses providing care at the two hospitals involved in the study”.

Please check Statistical section, page 7 lines 147-152.

**Point 6:** Table shows that the patients included some from the Orthopaedics and Surgery clinics- The authors need to clarify their patient sample description in the Methods Section (line 85)-Are these ‘medical’ patients?

**Response 6:** Thanks much. It is corrected and rewritten as:

The inclusion criteria were acute and chronically ill hospitalized patients for whom complete medical records were available and who were hospitalized for at least 7 days in a certain ward.

Please check page 4 line 78-79.

**Point 7:** To improve readability, authors should consider making the percentage proportions reported consistent by using uniform number of decimal values throughout the manuscript.

**Response 7:** Numbers and percentages throughout the manuscript were revised. Only 2 decimal values are used.

**Point 8:**  The authors have made many comparisons of their findings to those from similar studies elsewhere. However, I expected the authors to also expound the implications of their findings on the knowledge gaps in relation to those on rationality of thromboprophylaxis in this study.

**Response 8:** Thanks for your comments. It is revised and rewritten as

Thromboprophylaxis was underutilized in tertiary care hospitals in Northern Cyprus which denote a gap between evidence-based guideline and practice. By giving proper training to (HCPs) about DVT prevention and establishing a hospital wide clinical Pharmacist based DVT prevention program will decrease the morbidity and mortality associated with this disease process and will assure rational practices in North Cyprus.

Please check page 21 line 365-370.

**Point 9:** Was the sampling method ideal and was the sample size for the different phases adequate to draw conclusions? This needs to be considered.

**Response 9:** Thanks for your comments.

Yes, we think in terms of the settings of the study, as a representative sample size was enrolled. But as only 2 hospitals were chosen as study setting, we may not be able to generalize the study findings over all hospitals in North Cyprus. This is stated in the limitations of the study. See page 21 line 375-376.

**Point 10:** Both study sites are university (teaching) hospitals. Could this have affected the findings on knowledge levels of physicians/nurses?

Response 10: Thanks for your comment. The following lines were added into the limitations section, see page 21 line 376-386.

Both studied hospitals were teaching hospitals, in which healthcare professionals provide beside complex care; clinical education and training to current and future health professionals through educational and mentoring activities (31). Teaching hospitals tend to be early adopters of new evidence and technologies which leads to better outcomes and less mortality compared to non-teaching hospitals (32). Healthcare providers in teaching hospitals are more exposed to learning and teaching activities beside their preceptorship which encourages them to be theoretically and practically prepared for the role, adhere more closely to clinical policies, best practices and deliver a high quality care and services as a role model (33). This may further suggest inferior knowledge and practice of DVT prophylaxis in other settings with less teaching and mentorship, which necessitate further research and comparison to reach such a conclusion.

**Point 11:** Also, whilst the authors assessed nurse and physicians’ knowledge on DVT, it is not clear if the HCWs are aware of the Caprini’s RAM and if this is standard of practice. Is it possible that the low utilization/rationalization of thromboprophylaxis could have been due to lack of awareness?

**Response 11:** Thanks for your comment. We agree with the reviewer that a lack of awareness is likely to led to the study findings. In the implications and study recommendations section we recommended the following evidence-based interventions to raise awareness and improve thrombophylactic practice in North Cyprus hospitals.

An interventional programme that incorporates both education and a daily individual assessment of DVT risk factors is needed with an enclosed prophylaxis policy. The establishment of an effective deep venous thrombosis prophylaxis strategy in health care settings with evidence-based recommendations may be useful to improve patient safety, quality of life and best practices. Clinical pharmacists can utilize the Caprini risk assessment tool and assist health care providers in the rational implementation of the rational use of medications and antithrombotic prophylaxis in hospitals. Investments in training health care providers about deep venous thrombosis prophylaxis are needed to achieve the proper utilization of anti-thrombotic medications, this public health issue and regular medication errors related to inappropriate anticoagulant use deserve further consideration to decrease morbidity and mortality.

Please check page 22 line 390-400.

Thanks much for your effort and thus contribution to our work.
