# Supplementary material for: The rational use of thromboprophylaxis therapy in hospitalized patients and the perspectives of health care providers in Northern Cyprus
Source: PLoS One. 2020 Jul 15;15(7):e0235495. doi: 10.1371/journal.pone.0235495 (PMC7363080; doi:10.1371/journal.pone.0235495)
Supplement: Attachment — Submitted filename: Response to Reviewers.docx [file pone.0235495.s006.docx]

**Response to Reviewers Comments**

Dear reviewers and academic editor, thanks much for your time and fruitful comments on the article “The rational use of thromboprophylaxis therapy in hospitalized patients and the perspectives of health care providers in Northern Cyprus” Your review and comments would further help enhance the readability and the delivering of the message this article carries.

In the following few lines we would like to share our responses and describe the enhancement we made on the article following your comments and recommendations.

**Regarding first reviewer comments:**

**Point 1**: The sample size calculation is still not clear. Authors reported using 50% response distribution as one of the parameters in the sample size calculation. What was this response referring to? Is this a justifiable way of sample size calculation?

Response 1: Thanks for the comment. The sample size calculation is explained accordingly.

Sample size was calculated using Raosoft online sample size calculator. The calculation was based on 50 % response distribution, 5 % margin of error and 95 % confidence interval. The online software foundation is based on widely utilized descriptive studies sample size estimation formula proposed in Scott A. & Smith TM work (1) also cited by Satian et al (2).

Setting the response distribution to 50% is the most conservative assumption (3). The assumption that the response rate is 50 % was based on the idea that both responses and response rates were completely unknown since there are no previously published similar studies from North Cyprus. The calculated sample size was 172 patients, 98 physicians and 169 nurses. To ensure accuracy, the sample size was increased to 180 patients, 109 physicians and 237 nurses to account for any missing data or non-response rate. References (4 and 5) below cite examples of recent evidence adopting similar method in sample size determination.

1. Scott A, Smith TM.Estimation in multi-stage surveys. Journal of the American Statistical Association.1969;64(327):830-840.16.
2. Sathian B, Sreedharan J, Baboo SN, Sharan	K, Abhilash	E, Rajesh	E.Relevance of sample size determination in medical	research. Nepal	Journal	of Epidemiology.2010;1(1):4-10.
3. Raosoft Inc. Sample size calculator [<http://www.raosoft.com/samplesize.html>].
4. Abdo, Mahmoud Saeed, Mohamed Anwar Hammad, Sabariah Noor Harun, Tajunisah Mohamed Eusoff, and Siti Maisharah Sheikh Ghadzi. "Evaluation of knowledge, attitude and practice of healthcare providers towards life-threatening drug-drug interactions in Penang General Hospital, Malaysia." *Clinical Epidemiology and Global Health* (2020).
5. Zyoud, S.H., Abu Taha, A., Araj, K.F. *et al.* Parental knowledge, attitudes and practices regarding antibiotic use for acute upper respiratory tract infections in children: a cross-sectional study in Palestine. *BMC Pediatr* **15,** 176 (2015). <https://doi.org/10.1186/s12887-015-0494-5>.

**Point 2:** Mean and median still appear in table 3. Is there a need for continuous variables to be described/ summarised in both ways?

**Response 2:** Thanks for the comment, from Table 3 mean and standard deviation (SD) were removed. The table is revised and rewritten following your constructive comments. Please check Table 3, page 14.

**Point 3:**  In table 3: How were the comparisons made? were mean or median used for the comparisons of continuous variables?

**Response 3:** Thanks for the comment, from Table 3 mean and standard deviation (SD) were removed, as the non-parametric assumption was adopted comparisons were made based on median and IQR.

Please check Table 3, page 14.

**Point 4:** The Mann-Whitney U test and the Kruskal-Wallis tests were reported as the methods used to compare differences across groups. One would then assume that the data were deemed as being asymmetrical. Yet, mean (and SD) were compared as seen in the texts (line 264, 267, 270, 272. etc)

**Response 4:** Thanks for the comment, As the Mann-Whitney U test and the Kruskal-Wallis tests were used to compare differences across groups, Therefore, Mean & SD were deleted, and all values were changed to medians and rewritten as follow:

The median for practice on DVT prevention for nurses >31 years old was significantly lower than the median of nurses aged from 26-30 years and <25 years, (17), (18) and (21) (p<0.05), respectively. Meanwhile, the median for the general knowledge of DVT attained by nurses >31 years old was significantly higher than the median of nurses aged from 26-30 years and < 25 years old, (5), (4) and (4) (p<0.05), respectively.

Please check page 14 lines 262-282.

Thanks much for your effort and thus contribution to our work.
