# Supplementary material for: The rational use of thromboprophylaxis therapy in hospitalized patients and the perspectives of health care providers in Northern Cyprus
Source: PLoS One. 2020 Jul 15;15(7):e0235495. doi: 10.1371/journal.pone.0235495 (PMC7363080; doi:10.1371/journal.pone.0235495)
Supplement: Attachment — Submitted filename: Response to Reviewers.docx [file pone.0235495.s007.docx]

**Response to Reviewers Comments**

Dear reviewers and academic editor, thanks much for your time and fruitful comments on the article “The rational use of thromboprophylaxis therapy in hospitalized patients and the perspectives of health care providers in Northern Cyprus” Your review and comments would further help enhance the readability and the delivering of the message this article carries.

In the following few lines we would like to share our responses and describe the enhancement we made on the article following your comments and recommendations.

**Regarding second reviewer comments:**

**Point 1**: Abstract: Ensure you use a uniform tense throughout the abstract. The 'Aims' section is stated in present tense.

Response 1: Thanks for the comment. The abstract section is revised, and a uniform tense is used throughout the abstract following your recommendation and is rewritten as:

**Background:** Despite the presence of effective strategies and standard guidelines for the prevention of deep vein thrombosis (DVT), a considerable proportion of patients at risk of developing thromboembolism did not receive prophylaxis during hospitalization, while others received it irrationally, thus led to unwanted side effects.

**Aims:** This study aimed to evaluate the current thromboprophylaxis practice and management of hospitalized patients at risk of developing DVT, along with the assessment of health care providers (HCPs) knowledge, and attitudes regarding DVT prevention.

Please check line 16-22, page 1.

**Point 2:** Ensure that the proportions mentioned here match those in the results and discussion sections

Discussion:

Line313

"Indeed, after assessing 180 patients using the Caprini risk assessment tool, finding of the current study show that thromboprophylaxis regimens were appropriately provided to only approximately 52.2% of patients, consistent with the studies by White RH et al..."

The percentage proportion stated here does not add up with that stated in the abstract (47.7%).The authors should consider correcting this statement to reduce its ambiguity .

**Response 2:** Thanks for the comment, to match the proportions both in results and discussion section, the results and discussion section was revised, and the following line were added in the results section.

52.2% were identified as rationally managed,

Please check line 30, page 2.

**Point 3:** Conclusion:

There is some repetition in the authors conclusion. It needs to be made more succinct and focused on their study.

**Response 3:** Thanks for the comment, the conclusion section is revised, and the following line were deleted from the conclusion section and is rewritten as:

Despite the presence of effective strategies and standard protocols for the treatment and prevention of deep venous thrombosis, anticoagulants were administered inappropriately.

Based on the findings of the present study and international reports, adherence to VTE prophylaxis is still low in practice, a high level of irrationality in thromboprophylaxis therapy of hospitalized patients, and inappropriate administration of anticoagulants was observed.

Please check line 399, page 22.

Thanks much for your effort and thus contribution to our work.
